# Final Height and Cardiometabolic Outcomes in Young Adults with Very Low Birth Weight (<1500 g)

**DOI:** 10.1371/journal.pone.0112286

**Published:** 2014-11-14

**Authors:** Ryosuke Sato, Masato Maekawa, Rieko Genma, Kenji Shirai, Shigeru Ohki, Hiroshi Morita, Takafumi Suda, Hiroshi Watanabe

**Affiliations:** 1 Department of Internal Medicine II, Hamamatsu University School of Medicine, Hamamatsu, Japan; 2 Department of Endocrinology and Metabolism, Seirei Hamamatsu General Hospital, Hamamatsu, Japan; 3 Department of Laboratory Medicine, Hamamatsu University School of Medicine, Hamamatsu, Japan; 4 Department of Neonatology, Seirei Hamamatsu General Hospital, Hamamatsu, Japan; 5 Department of Clinical Pharmacology and Therapeutics, Hamamatsu University School of Medicine, Hamamatsu, Japan; Virgen Macarena University Hospital, School of Medicine, University of Seville, Spain

## Abstract

**Objective:**

Individuals with very low birth weight (VLBW; <1500 g) are known to be predisposed to both short final height and cardiometabolic disorders. However, associations between final height and cardiometabolic outcomes including glucose metabolism in VLBW individuals in young adulthood are not fully investigated.

**Methods:**

We investigated glucose metabolism and other cardiometabolic outcomes such as lipid profiles, blood pressure, renal function, urinary albumin, and thyroid function in young adults with VLBW born between 1980 and 1990. Short stature was defined as a final height <10th percentile. Glucose intolerance [diabetes, impaired glucose tolerance (IGT), and impaired fasting glucose (IFG)] was determined using 75-g oral glucose tolerance tests. Associations between final height and cardiometabolic outcomes were examined using logistic or multiple linear regression.

**Results:**

A total of 628 VLBW individuals were screened and 111 young adults with VLBW (19–30 years) participated in the study. Of the participants, 40 subjects (36%) had short stature with a final height <10th percentile. Eight subjects (7.2%) had glucose intolerance (1, diabetes; 6, IGT; 1, IFG). Short stature was correlated with glucose intolerance (odds ratio 11.1; 95% CI 1.92, 99.7; *P* = 0.006). Final height was inversely associated with the homeostatic model assessment (HOMA) of insulin resistance, HOMA-β, insulinogenic index, and total/LDL-cholesterol. The associations of final height with insulin sensitivity and lipid profiles remained after adjustment for target height and age at puberty onset.

**Conclusions:**

Shorter final height was associated with less favorable metabolic profiles in young adults with VLBW, and may be partly mediated by reduced insulin sensitivity. These associations were independent of target height or age at puberty onset.

## Introduction

Neonatal intensive care has markedly improved the survival rate of very low birth weight infants (VLBW; birth weight <1500 g) in recent decades [1], [2]. The first generation of VLBW infants who survived because of improvements in neonatal care are now in their twenties or thirties. Therefore, we are now seeing a considerable number of VLBW infants reaching young adulthood, and the potential delayed effects of VLBW on later life have emerged as a significant concern. Several epidemiological studies have shown associations between low birth weight (<2500 g) and onset of type 2 diabetes and cardiovascular disease in later life [3]–[5]. Regarding pathophysiological pathways linking low birth weight to cardiometabolic outcomes, it is proposed that fetal malnutrition in the gestational period, which prevents appropriate fetal growth in utero, establishes thrifty phenotype in premature babies. This phenotype is considered to predispose them to cardiometabolic disorders later in life [6]. However, few studies [7], [8] have investigated glucose metabolism in young adults with VLBW. Thus, the clinical factors affecting glucose metabolism in VLBW individuals are still unclear.

Previous studies have shown that short final height in adulthood is associated with the risk of type 2 diabetes [9], [10] and cardiovascular disease [11]. Short final height in individuals born prematurely is considered to be a long-term consequence of intrauterine retardation and catch-up failure in childhood, which may be also associated with cardiometabolic risk [12]. In fact, VLBW individuals are known to have both shorter statures and higher glucose levels compared with term subjects [7]. If final height serves as an index of cardiometabolic outcomes in VLBW individuals, VLBW individuals with a short final height could be encouraged to undergo medical checkups in early adulthood. Therefore, we conducted an observational study of young adults with VLBW to clarify the associations between final height and cardiometabolic outcomes, including glucose metabolism, lipid profiles, blood pressure, renal function, urinary albumin, and thyroid function, which are associated with cardiometabolic disorders [13]. To investigate glucose metabolism in detail, we performed oral glucose tolerance tests (OGTTs), which enabled us to assess the potential mechanism of glucose intolerance. Final height is influenced by genetic factors [14], [15] and sexual maturation [16], in addition to poor fetal environmental conditions. For this reason, we also extended the analysis to validate the associations between final height and cardiometabolic outcomes in a model that included clinical factors of genetic inheritance and sexual maturation.

## Subjects and Methods

### Study population and procedures

We conducted an observational study of young adults with VLBW who were born between 1980 and 1990 and treated at the neonatal intensive care unit at Seirei Hamamatsu General Hospital (Hamamatsu, Japan). Further details of this study setting were previously reported [8]. The neonatal intensive care unit was established in 1977 and more than 400 neonates are treated annually. The hospital's birth database was used to identify VLBW individuals and we excluded subjects who were dead or suffered from severe neurodevelopmental impairment. We wrote to the remaining individuals to invite them to participate in the study in May 2010. Anthropometric and cardiometabolic assessments, including a standard 75-g OGTT, were performed at Seirei Hamamatsu General Hospital between July 2010 and March 2011. After completing these assessments, we conducted extended analyses to validate the associations between final height and cardiometabolic outcomes taking into account the effects of genetic inheritance and sexual maturation on final height. We sent the participants questionnaires regarding their parents' heights and the age at puberty onset. Parental heights are an index of genetic inheritance and were used to calculate the subjects' target height. Age at puberty onset was used as an index of sexual maturation.

VLBW was defined as a birth weight of <1500 g and extremely low birth weight (ELBW) was defined as a birth weight of <1000 g according to World Health Organization criteria. Small for gestational age (SGA) status was defined as a birth weight <10th percentile for gestational age according to standards developed by a study group of the Japanese Health Ministry [17]. Birth weight ≥10th percentile for gestational age was defined as appropriate for gestational age (AGA).

The study was approved by the ethical committee of Seirei Hamamatsu General Hospital. All participants provided written informed consent to participate in the study.

### Measurements and calculations

The anthropometric and cardiometabolic assessments in young adulthood were performed at Seirei Hamamatsu General Hospital. Height was measured using a stadiometer to the nearest millimeter. Short stature was defined as a final height of <10th percentile based on Japanese population data published by the Japanese Ministry of Education, Culture, Sports, Science, and Technology [18]. Subjects with a final height ≥10th percentile were included in a non-short stature group. In the extended analyses, target height was calculated as follows [19]: males, (paternal height + maternal height +13)/2+2; females, (paternal height + maternal height – 13)/2+2. Growth failure was defined as a final height <10th percentile and below the target height. The age at puberty onset was defined as the earliest onset age of voice changes, growth of pubic hair, or penis and testicle development in males and as the earlier onset age of menarche or growth of pubic hair in females.

All participants underwent a standard 75-g OGTT after a 10-h overnight fast. Plasma glucose and serum insulin levels were measured during the OGTT. Fasting glucose levels and 2-h glucose levels were used for the diagnosis of diabetes, impaired glucose tolerance (IGT), and impaired fasting glycemia (IFG) according to the World Health Organization criteria [20]. Glucose intolerance was defined as diabetes, IGT, or IFG. Because 1 h plasma glucose concentration was reported to be associated with the future risk of type 2 diabetes and atherosclerosis, 1 h plasma glucose levels >8.6 mmol/l (155 mg/dl) in subjects without diabetes, IGT, or IFG was defined as isolated 1-h hyperglycemia [21], [22]. Plasma glucose and serum insulin levels were measured using hexokinase method and chemiluminescent enzyme immunoassay method, respectively, on an autoanalyzer (JCA-BM2250; JEOL, Tokyo, Japan). Fasting blood samples were used to measure total cholesterol, HDL-cholesterol, LDL-cholesterol, triglyceride, creatinine, thyroid stimulating hormone (TSH), and free T4 (fT4). Urine samples were used to estimate the urinary albumin/creatinine ratio. Glycated hemoglobin A1c (HbA1c) was measured using a high-performance liquid chromatography (HPLC) method on an automated glycohemoglobin analyzer (HLC-723G8; Tosoh Bioscience, Tokyo, Japan). TSH >2.5 mU/l, which was reported to be associated with future hypothyroidism in a large-scale longitudinal cohort study [23], was defined as elevated TSH.

Insulin sensitivity was estimated using the homeostatic model of assessment for insulin resistance (HOMA-IR) [24]: fasting insulin (µU/ml) × fasting glucose (mmol/l)/22.5. Pancreatic β cell function was estimated using the homeostatic model of assessment for β cell (HOMA-β) and insulinogenic index. HOMA-β was calculated as follows [24]: 20× fasting insulin (µU/ml)/[fasting glucose (mmol/l) −3.5]. Insulinogenic index, the index of early insulin response during OGTT, was calculated as follows: Δinsulin (30 min–0 min)/Δglucose (30 min–0 min). The estimated glomerular filtration rate (eGFR) was calculated using the formula developed by the Japanese Society of Nephrology [25]: eGFR (ml/min/1.73 m^2^)  = 194×Cre^−1.094^× Age^−0.287^ (×0.739 for females).

### Statistical analysis

Statistical analyses were conducted using SPSS software version 20.0 (IBM, Inc., Armonk, NY, USA). Quantitative variables are expressed as the mean and SD or 95% CI. Categorical variables are presented as the number and percentage. The Shapiro–Wilk test was used to assess the normality of each variable. Continuous variables were compared between subgroups of subjects using Student's *t* test or the Mann–Whitney *U* test. Categorical variables were compared between subgroups of subjects using Pearson's χ^2^ test or Fisher's exact test. Logistic regression was used to evaluate the association between short stature and glucose intolerance with adjustment for sex, body weight, family history of diabetes (including second-degree relatives), and SGA/AGA. The association between short stature and a combined category of glucose intolerance and isolated 1-h hyperglycemia was also assessed with adjustment for the same factors. Multiple linear regression was used to investigate the associations between final height and cardiometabolic outcomes. Sex, body weight, family history of diabetes, and SGA/AGA were also included in the model for the adjustment. In the extended analyses performed to validate the associations of final height with cardiometabolic outcomes, the multiple linear regression model was adjusted for sex, body weight, family history of diabetes, SGA/AGA, target height, and age at puberty onset. This model was also used to determine correlations between age at puberty onset and cardiometabolic outcomes. Multiple linear regression was also used to evaluate the associations between growth failure and cardiometabolic outcomes, with adjustment for sex, body weight, family history of diabetes, SGA/AGA, and age at puberty onset. In all analyses, values of *P*<0.05 were considered statistically significant.

## Results

### Subject characteristics

**Figure 1:**
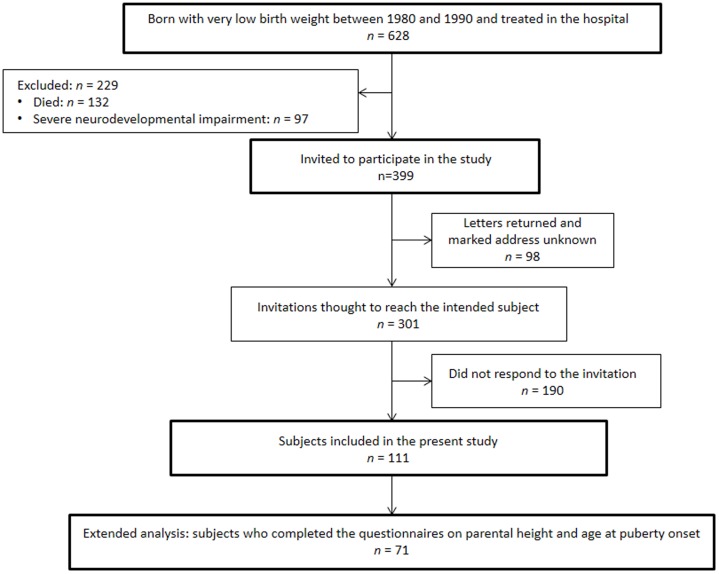
Subject disposition.

A total of 628 Japanese VLBW individuals were born between 1980 and 1990, and were treated at the neonatal intensive care unit (Figure 1). Of these, 229 were excluded because of death (*n* = 132) or severe neurodevelopmental impairment (*n* = 97). We sent invitations to the remaining 399 subjects, of which 98 were returned marked as address unknown. Of the 301 individuals who were thought to have received the letters, 111 subjects aged 19–30 years participated in the study. The characteristics of these subjects are summarized in Table 1. Of the 111 participants, 71 completed the questionnaires regarding parental height and age at puberty onset. Therefore, the extended analyses were conducted in this group of 71 subjects.

**Table 1:** Clinical characteristics of the subjects according to final height.

	Total	Final height		*P* *
	(*n* = 111)	<10th percentile	≥10th percentile	
		(*n* = 40)	(*n* = 71)	
Female, *n* (%)	69 (62.2)	18 (45.0)	51 (71.8)	0.005
**Infant/childhood variables**				
Gestational age (weeks)	29.7 (3.1)	29.9 (3.3)	29.6 (3.0)	0.769
Birth weight (g)	1152 (235)	1072 (240)	1198 (221)	0.007
SGA†, *n* (%)	39 (35.1)	20 (50.0)	19 (26.8)	0.014
ELBW, *n* (%)	33 (29.7)	19 (47.5)	14 (19.7)	0.002
Multiple pregnancy, *n* (%)	18 (16.2)	6 (15.0)	12 (16.9)	0.794
GH treatment, *n* (%)	2 (1.8)	2 (5.0)	0 (0.0)	0.128
**Adulthood variables**				
Age (years)	24.8 (3.0)	24.9 (3.3)	24.8 (2.9)	0.953
Height (cm)				
Male	163.7 (6.4)	158.8 (3.6)	169.1 (3.8)	<0.001
Female	154.7 (5.7)	147.6 (3.6)	157.3 (3.9)	<0.001
Body weight (kg)				
Male	54.9 (8.8)	51.3 (9.0)	58.9 (6.9)	0.006
Female	50.7 (10.5)	47.9 (14.9)	51.6 (8.5)	0.015
BMI (kg/m^2^)	20.9 (3.8)	21.0 (4.9)	20.8 (3.0)	0.414
BMI>25 kg/m^2^, *n* (%)	15 (13.5)	8 (20.0)	7 (9.9)	0.133
Smoking, *n* (%)	37 (33.3)	11 (28.2)	26 (36.1)	0.328
Family history of diabetes, *n* (%)	22 (19.8)	7 (18.0)	15 (20.8)	0.645
Glucose intolerance, *n* (%)	8 (7.2)	6 (15.0)	2 (2.8)	0.025
Diabetes	1	0	1	
IGT	6	5	1	
IFG	1	1	0	
Isolated 1-h hyperglycemia‡, *n* (%)	13 (11.7)	5 (12.5)	8 (11.3)	0.536
Plasma glucose (mmol/l)§				
Fasting	5.04 (0.43)	5.05 (0.48)	5.03 (0.40)	0.444
1 h	6.81 (1.91)	6.97 (2.12)	6.73 (1.80)	0.719
2 h	5.80 (1.35)	5.82 (1.31)	5.79 (1.38)	0.949
HbA1c (%)	5.39 (0.31)	5.36 (0.34)	5.41 (0.29)	0.407
HOMA-IR	1.53 (1.14)	1.69 (1.69)	1.43 (0.65)	0.476
HOMA-β	91.7 (61.2)	100.8 (88.4)	86.6 (38.2)	0.606
Insulinogenic index	23.8 (23.0)	26.5 (28.6)	22.2 (19.2)	0.432
Cholesterol (mg/dl)				
Total	184.8 (31.3)	192.3 (38.8)	180.5 (25.6)	0.104
LDL	104.3 (28.4)	109.0 (34.9)	101.6 (24.0)	0.346
HDL	65.7 (13.3)	66.4 (14.9)	65.3 (12.4)	0.673
Triglycerides (mg/dl)	81.8 (72.2)	92.0 (91.6)	76.0 (58.5)	0.789
Blood pressure (mmHg)				
Systolic	118 (15.5)	117.2 (14.7)	118.6 (16.1)	0.665
Diastolic	70 (11.1)	68.4 (11.8)	70.2 (10.7)	0.417
Creatinine (mg/dl)	0.66 (0.13)	0.69 (0.14)	0.64 (0.12)	0.024
eGFR (ml/min/1.73 m^2^) ||	104.6 (18.4)	104.8 (21.4)	104.5 (16.6)	0.705
Urinary albumin/creatinine (mg/g)	16.8 (41.0)	10.6 (17.4)	20.1 (48.8)	0.763
TSH (mU/l)	1.41 (0.68)	1.51 (0.78)	1.35 (0.62)	0.340
TSH>2.5 mU/l, *n* (%)	9 (8.1)	5 (12.5)	4 (5.6)	0.279
fT4 (ng/dl)	1.16 (0.15)	1.13 (0.13)	1.17 (0.16)	0.344

Data are expressed as the mean (SD) or *n* (%).

*P values are shown for between-group comparisons.

† Defined as birth weight <10th percentile for gestational age according to the standards proposed by a study group of the Japanese Health Ministry.

‡ Defined as 1-h plasma glucose levels>8.6 mmol/l (155 mg/dl) without diabetes, IGT, or IFG.

§ Measured during 75-g oral glucose tolerance tests. The reference ranges of glucose levels according to the World Health Organization standards are as follows: fasting <6.1 mmol/l (110 mg/dl); and 2-h after a glucose load <7.8 mmol/l (140 mg/dl).

|| eGFR = 194×Cre^−1.094^× Age^−0.287^ (×0.739 for females).

eGFR, estimated glomerular filtration rate; ELBW, extremely low birth weight (<1000 g); fT4, free T4; GH, growth hormone; SGA, small for gestational age; TSH, thyroid stimulating hormone.

Of the 111 subjects included in this study, 40 (36%) had a short stature with a final height <10th percentile, comprising the short stature group. We compared the characteristics of the short stature group and the non-short stature group. Birth weight, height in adulthood, and body weight in adulthood were significantly lower in the short stature group than in the non-short stature group. SGA and ELBW were significantly more frequent in the short stature group than in the non-short stature group. There were fewer females in the short stature group than in the non-short stature group. Only two subjects were treated with growth hormone (GH) in childhood; both were in the short stature group. The proportion of subjects with high body mass index (>25 kg/m^2^) in adulthood tended to be greater in the short stature group than in the non-short stature group.

### Associations between final height and cardiometabolic outcomes

The cardiometabolic characteristics of the subjects in young adulthood, including glucose metabolism, lipid profiles, blood pressure, renal function, urinary albumin, and thyroid function, are summarized in Table 1. The proportions of subjects with glucose intolerance (diabetes, IGT, or IFG) (15.0% vs. 2.8%, *P* = 0.025) or a combined category of glucose intolerance and isolated 1-h hyperglycemia (27.5% vs. 14.1%, *P* = 0.083) were higher in the short stature group. The total cholesterol level tended to be higher in the short stature group than in the non-short stature group.

Logistic regression with adjustment for sex, body weight, family history of diabetes, and SGA/AGA revealed that short stature was significantly associated with glucose intolerance (adjusted odds ratio [OR] 11.1; 95% CI 1.92, 99.7; *P* = 0.006) and a combined category of glucose intolerance and isolated 1-h hyperglycemia (adjusted OR 3.55; 95% CI 1.13, 12.1; *P* = 0.030).

We next investigated the associations between final height and cardiometabolic outcomes using multiple linear regression (Table 2) with adjustment for sex, body weight, family history of diabetes, and SGA/AGA. In these analyses, final height in adulthood was negatively associated with HOMA-IR, HOMA-β, insulinogenic index, total cholesterol, and LDL-cholesterol. Final height also showed trends towards negative associations with fasting glucose and 2-h glucose, and a trend towards a positive association with HDL-cholesterol. Final height was not associated with blood pressure, renal function, urinary albumin, or thyroid function.

**Table 2:** Associations between final height and cardiometabolic outcomes in young adults with VLBW in the primary and extended analyses.

Outcome variable	Primary analyses*		Extended analyses†	
	(*n* = 111)		(*n* = 71)	
	β (95% CI)	*P*	β (95% CI)	*P*
Plasma glucose (mmol/l) ‡				
Fasting	−0.012 (−0.026, 0.002)	0.083	−0.027 (−0.049, −0.005)	0.016
1 h	−0.032 (−0.096, 0.032)	0.320	−0.063 (−0.167, 0.041)	0.228
2 h	−0.036 (−0.081, 0.009)	0.118	−0.054 (−0.131, 0.024)	0.171
HbA1c (%)	−0.004 (−0.015, 0.007)	0.469	−0.013 (−0.029, 0.004)	0.134
HOMA-IR	−0.080 (−0.109, −0.051)	<0.001	−0.071 (−0.111, −0.032)	<0.001
HOMA-β	−3.291 (−5.090, −1.492)	<0.001	−1.691 (−3.966, 0.584)	0.142
Insulinogenic index	−1.005 (−1.798, −0.212)	0.014	−0.155 (−1.298, 0.988)	0.787
Cholesterol (mg/dl)				
Total	−1.068 (−2.122, −0.013)	0.047	−1.519 (−3.363, 0.324)	0.105
LDL	−1.130 (−2.024, −0.236)	0.014	−1.935 (−3.430, −0.440)	0.012
HDL	0.341 (−0.081, 0.764)	0.112	0.732 (−0.001, 1.465)	0.050
Triglycerides (mg/dl)	−0.556 (−2.970, 1.858)	0.649	0.331 (−4.291, 4.953)	0.887
Blood pressure (mmHg)				
Systolic	−0.197 (−0.691, 0.297)	0.430	−0.134 (−0.842, 0.574)	0.707
Diastolic	−0.035 (−0.396, 0.327)	0.850	0.169 (−0.352, 0.690)	0.519
eGFR (ml/min/1.73 m^2^)§	−0.401 (−1.066, 0.264)	0.235	−0.329 (−1.347, 0.690)	0.521
Urinary albumin/creatinine (mg/g)	1.078 (−0.458, 2.614)	0.167	1.388 (−1.292, 4.068)	0.304
TSH (mU/l)	−0.013 (−0.037, 0.011)	0.278	−0.009 (−0.044, 0.027)	0.638
fT4 (ng/dl)	0.003 (−0.003, 0.008)	0.329	0.002 (−0.007, 0.011)	0.654

*Associations were adjusted for sex, body weight, family history of diabetes, and SGA/AGA.

† Associations were adjusted for sex, body weight, family history of diabetes, SGA/AGA, target height, and age at puberty onset.

‡ Measured during 75-g oral glucose tolerance tests.

§ eGFR = 194×Cre^−1.094^× Age^−0.287^ (×0.739 for females).

AGA, appropriate for gestational age; CI, confidence interval; eGFR, estimated glomerular filtration rate; fT4, free T4; SGA, small for gestational age; TSH, thyroid stimulating hormone.

### Associations of final height, growth failure, and age at puberty onset with cardiometabolic outcomes in the extended analyses

We conducted extended analyses in 71 responders (Figure 1) to validate the associations between final height and cardiometabolic outcomes. There were no significant differences in clinical characteristics between these 71 subjects and the 40 subjects excluded from the extended analyses. The parental heights, target height, growth failure rate and age at puberty onset of the males and the females are shown in Table 3. The proportion of subjects whose final height was below the target height was not significantly different between males and females (*P* = 0.507). However, the proportions of subjects with a final height <10th percentile (*P* = 0.006) or those with growth failure (i.e., final height below the target height and <10th percentile; *P* = 0.022) were both significantly greater in males than in females.

**Table 3:** Parental height, target height, and age at puberty onset of subjects with VLBW who were included in the extended analyses.

	Males	Females	*P* *
	(*n* = 23)	(*n* = 48)	
Paternal height (cm)	167.4 (5.45)	168.9 (5.96)	0.287
Maternal height (cm)	155.8 (3.33)	156.2 (4.95)	0.681
Target height (cm)	169.0 (5.55)	158.1 (4.53)	<0.001
Below target height, *n* (%)	19 (82.6)	34 (70.8)	0.507
<10th percentile, *n* (%)	14 (60.9)	13 (27.1)	0.006
Growth failure, *n* (%)†	12 (52.2)	11 (22.9)	0.022
Age at puberty onset (years)	13.1 (1.50)	12.0 (1.68)	0.006

Data are expressed as the mean (SD) or *n* (%).

*P values are shown for the comparison between males and females.

† Below the target height and <10th percentile.

Table 2 shows the associations between final height and cardiometabolic outcomes in the extended analysis, with adjustment for sex, body weight, family history of diabetes, SGA/AGA, target height, and age at puberty onset. Final height was negatively associated with fasting glucose and HOMA-IR, but was not associated with HOMA-β or the insulinogenic index. The associations between final height and lipid profiles in the extended analysis were similar to those in the primary analysis. Final height was negatively associated with LDL-cholesterol. Final height tended to be negatively associated with total cholesterol and tended to be positively associated with HDL-cholesterol.

We also evaluated the associations between growth failure and cardiometabolic outcomes in cohort of 71 subjects using multiple linear regression (Table 4) with adjustment for sex, body weight, family history of diabetes, SGA/AGA, and age at puberty onset. Growth failure was positively associated with HOMA-IR, and tended to have a positive association with 1-h plasma glucose, but was not associated with HOMA-β or the insulinogenic index. Growth failure was not associated with lipid profiles, blood pressure, renal function, urinary albumin, and thyroid function.

**Table 4:** Associations of growth failure and age at puberty onset with cardiometabolic outcomes in young adults with VLBW in the extended analyses.

	Growth failure*		Age at puberty onset (yr)†	
Outcomes	β (95% CI)	*P*	β (95% CI)	*P*
Plasma glucose (mmol/l)‡				
Fasting	0.044 (−0.080, 0.168)	0.481	−0.009 (−0.084, 0.067)	0.819
1 h	0.551 (−0.017, 1.118)	0.057	0.268 (−0.091, 0.627)	0.141
2 h	0.242 (−0.180, 0.665)	0.256	0.127 (−0.140, 0.394)	0.344
HbA1c (%)	0.011 (−0.081, 0.104)	0.804	0.081 (0.024, 0.139)	0.006
HOMA-IR	0.247 (0.020, 0.474)	0.033	0.074 (−0.061, 0.209)	0.276
HOMA-β	9.445 (−2.953, 21.84)	0.133	4.333 (−3.516, 12.18)	0.274
Insulinogenic index	−1.683 (−7.914, 4.548)	0.591	0.741 (−3.203, 4.686)	0.708
Cholesterol (mg/dl)				
Total	5.564 (−4.638, 15.77)	0.280	2.769 (−3.591, 9.129)	0.387
LDL	5.333 (−3.252, 13.92)	0.219	2.772 (−2.385, 7.929)	0.287
HDL	−2.047 (−6.143, 2.049)	0.322	−0.473 (−3.002, 2.056)	0.710
Triglycerides (mg/dl)	9.928 (−15.05, 34.91)	0.430	0.785 (−15.16, 16.73)	0.922
Blood pressure (mmHg)				
Systolic	−2.125 (−5.935, 1.686)	0.269	2.328 (−0.114, 4.769)	0.061
Diastolic	−2.250 (−5.031, 0.530)	0.111	1.172 (−0.625, 2.968)	0.197
eGFR (ml/min/1.73 m^2^) §	0.183 (−5.375, 5.741)	0.948	−0.347 (−3.860, 3.166)	0.844
Urinary albumin/creatinine (mg/g)	1.325 (−13.37, 16.02)	0.858	0.606 (−8.639, 9.851)	0.896
TSH (mU/l)	0.068 (−0.128, 0.264)	0.493	0.081 (−0.043, 0.205)	0.194
fT4 (ng/dl)	−0.023 (−0.073, 0.027)	0.370	0.011 (−0.021, 0.043)	0.482

*Growth failure was defined as a final height <10th percentile and below the target height. Associations were adjusted for sex, body weight, family history of diabetes, SGA/AGA, and age at puberty onset.

† Associations were adjusted for sex, height, body weight, family history of diabetes, SGA/AGA and target height.

‡ Measured during 75-g oral glucose tolerance tests.

§ eGFR = 194×Cre^−1.094^× Age^−0.287^ (×0.739 for females).

AGA, appropriate for gestational age; CI, confidence interval; eGFR, estimated glomerular filtration rate; fT4, free T4; SGA, small for gestational age; TSH, thyroid stimulating hormone.

Multiple linear regression showed that age at puberty onset was positively associated with HbA1c after adjusting for sex, height, body weight, family history of diabetes, SGA/AGA, and target height. However, the age at puberty onset was not associated with other glucose metabolism-related variables. The age at puberty onset was significantly associated with HbA1c in males (β = 0.098; 95% CI 0.003, 0.194; *P* = 0.044) but not in females (β = 0.079; 95% CI −0.007, 0.166; *P* = 0.070).

### Exploratory analysis of an association between SGA and thyroid function in young adulthood

Of 111 subjects enrolled in the study, 39 were born SGA and 72 were born AGA. These subjects were used to examine an association between SGA and thyroid function. TSH levels tended to be higher in the SGA group than in the AGA group (1.64±0.14 mU/l vs. 1.29±0.06 mU/l, *P* = 0.100). In addition, the proportion of subjects with elevated TSH levels within the reference range (>2.5 mU/l) tended to be greater in the SGA group than in the AGA group (15.4% vs. 4.2%, *P* = 0.064). Multiple linear regression with adjustment for sex, body mass index, and smoking showed that SGA was positively associated with TSH levels in young adults with VLBW (β = 0.188; 95% CI 0.058, 0.318; *P* = 0.005). TSH levels were not significantly associated with cardiometabolic outcomes in multiple linear regression with adjustment for SGA/AGA (data not shown).

## Discussion

To our knowledge, this is the first study to show that short final height has unfavorable associations with glucose metabolism and lipid profiles in young adults with VLBW. There are three key findings of the present study: 1) hyperglycemia was relatively common in young adults with VLBW and short stature; 2) final height was negatively associated with glucose levels and HOMA-IR; and 3) final height was negatively associated with total cholesterol and LDL-cholesterol and tended to be positively associated with HDL-cholesterol.

Postnatal catch-up during infancy following intrauterine growth restriction has been shown to be associated with glucose intolerance in later life [26], [27]. However, the present study revealed that, in young adults with VLBW, final catch-up failure is a manifestation of co-existing cardiometabolic disorders, including glucose intolerance and dyslipidemia. A previous French study revealed that young adults born SGA were shorter and had higher glucose and insulin levels than those born AGA [12]. Studies of general populations have also shown that short final height in adulthood is associated with the risk of type 2 diabetes [9], [10]. Meanwhile, other recent studies indicated that short leg or arm length is associated with cardiometabolic disorders, including type 2 diabetes [28]–[30]. Thus, an imbalance in limb and trunk lengths might be a potential marker for cardiometabolic disorders, as well as final height in young adults with VLBW.

The unfavorable associations of short final height with glucose metabolism in young adults with VLBW seem to be mediated by reduced insulin sensitivity rather than β cell dysfunction. The association between final height and HOMA-IR was consistently observed in the primary analyses and in the extended analyses, although the associations of final height with HOMA-β or the insulinogenic index were inconsistent. The associations between final height and lipid profiles, which are related to insulin sensitivity [31], were generally consistent between the primary analyses and the extended analyses. It is notable that final height was negatively associated with HOMA-β and the insulinogenic index, indicating HOMA-β and insulinogenic index are higher in shorter subjects. In the extended analyses, growth failure was positively associated with HOMA-IR, but was not associated with the indices of β cell function. These findings indicate that final height has a greater association with insulin sensitivity than β cell function in young adults with VLBW. The reason for the negative association between final height and β cell function in the primary analysis is unclear. However, one explanation is that of greater β cell compensation for reduced insulin sensitivity in shorter subjects, which can lead to future β cell exhaustion [31], [32]. Therefore, it is possible that the findings may differ if glucose status is assessed in middle-aged subjects with VLBW.

In the extended analyses, multiple linear regression showed that the age at puberty onset was positively associated with HbA1c, especially in males. Previous studies have shown that early timing of puberty was associated with cardiometabolic disorders [33]–[36], although a recent large-scale population-based study reported that the age at menarche was not associated with diabetes [37]. Most of these studies included female subjects and investigated the associations between early menarche with cardiometabolic outcomes [35]–[37]. Therefore, the associations between male pubertal timing and cardiometabolic outcomes are still unclear. Our findings suggest that the impact of pubertal timing on glucose metabolism in individuals with VLBW differs between males and females, and might be due to the different effects of sex hormones on glucose metabolism. However, we cannot prove the difference of hormonal effects as sex hormones were not measured in the present study.

Thyroid function is also associated with cardiometabolic disorders [13]. In the present study, thyroid function was not significantly different between the short stature group and the non-short stature group. We then conducted exploratory analyses evaluating the association between SGA and TSH levels in young adulthood because a previous study showed that prepubertal children born SGA had higher TSH levels than those born AGA [38]. In the exploratory analyses, 9 subjects (8.1%) had elevated TSH levels (>2.5 mU/l), which was reported to be associated with future hypothyroidism in an earlier study [23], and SGA was positively associated with TSH levels in young adulthood. Thus, our results suggest that individuals with VLBW, especially those that are born SGA, may be predisposed to hypothyroidism in later life.

### Strengths and Limitations

Some strengths of our study include the relatively large number of subjects from the first generation of young adults with VLBW, who account for only 0.5% of the general population. These subjects underwent OGTTs, allowing us to assess markers for insulin sensitivity and β cell function. Therefore, we were able to assess the potential mechanism explaining the association between short stature and glucose intolerance. We also evaluated other cardiometabolic outcomes, including lipid profiles, blood pressure, renal function, and thyroid function. In addition, because this study was performed in a genetically homogenous population, it was not necessary to consider possible ethnic differences in cardiometabolic outcomes.

Some limitations of the study include the lack of information on growth trajectories in childhood and on maternal factors such as advanced age, smoking, gestational diabetes, hypertension, assisted reproductive technology and socioeconomic status. Furthermore, we did not enroll any control subjects, which limits our ability to evaluate the association between VLBW itself and cardiometabolic outcomes. In terms of the study outcomes, although we have demonstrated that short final height is associated with adverse glucose or lipid profiles in young adults with VLBW, we did not evaluate cardiovascular-related events. Moreover, a recent population-based study reported that height in mid-life was positively associated with mortality [39]. The findings of the present study should be carefully interpreted considering the limitation that the outcomes are surrogate markers for cardiovascular disease. In the extended analyses, we incorporated information regarding parental heights and the age at puberty onset, which were obtained by questionnaires. The age at puberty onset, in particular, may be subject to recall bias, although the extent of recall bias should be reduced in females because the age at menarche is a significant event [40]. Of 111 participants included in the primary analyses, 71 (64%) responded to the questionnaires and were included in the extended analyses. Although the response rate was not necessarily high, there were no significant differences in the baseline clinical characteristics between the responders and the non-responders.

## Conclusions

Shorter final height is unfavorably associated with glucose metabolism and lipid profiles in young adults with VLBW, and these associations might be mediated by reduced insulin sensitivity. Notably, the associations between final height and variables related to insulin sensitivity and lipid profiles were not confounded by target height or age at puberty onset. Our results suggest that final height is a potential indicator of cardiometabolic outcomes in individuals with VLBW and could support public health interventions. Further large-scale prospective studies are needed to confirm the association between shorter final height and cardiometabolic disorders that underlie cardiovascular disease in individuals with VLBW.
